# Identifying appropriate phase II trial designs

**DOI:** 10.1186/1745-6215-12-S1-A87

**Published:** 2011-12-13

**Authors:** Sarah R Brown

**Affiliations:** 1 Clinical Trials Research Unit, University of Leeds, Leeds, UK

## 

The phase II to III transition in the drug development process is associated with the highest risk compared to transition rates between other phases [1]. With increasing pressure to improve efficiency in this process it is essential that phase II trials are designed based on informed decisions, and to provide reliable results.

With over 120 different phase II trial designs available [2], identifying which designs are appropriate can be difficult and is based on a number of elements. Randomisation, endpoint selection, and statistical design all contribute to the decision as to which trial design to use. Additionally, in an environment of ever-changing treatments and newly developed biomarkers, it is vital that the way in which treatments may work is incorporated into the decision making process. As a solution to identifying designs, a structured thought process, guidance manual and library of phase II trial designs has been developed [2]. This considers key elements associated with identifying a phase II trial design, and is intended to facilitate interaction between the clinician and statistician, as well as providing a structured and systematic approach to identifying appropriate trial designs.

Challenges remain, however, in choosing between a number of designs identified that fit trial-specific design criteria. Researchers may consider practical elements of conducting a trial, or previous experience, to determine which design to use. However further consideration to the performance of different designs may be necessary. Simulation provides an ideal opportunity to evaluate this under differing trial scenarios, and is often used in the design of phase I trials.

An overview of the role of phase II trials in the drug development pathway will be presented, highlighting current issues and solutions to identifying appropriate trial designs, including a worked example. Further discussion will include the challenges in choosing between designs, with an example of the use of simulation to evaluate trial design presented.
